# Longitudinal surveillance of the molecular evolution of methicillin-resistant *Staphylococcus aureus* isolates from pediatric patients in Shanghai, China, from 2013 to 2022

**DOI:** 10.1128/msystems.00371-25

**Published:** 2025-04-30

**Authors:** Xiaozhou Pan, Jiao Zhang, Fen Pan, Chun Wang, Huihong Qin, Fangyuan Yu, Tiandong Zhang, Wenxin Liu, Qianyue Wu, Zhan Ma, Wenhao Weng, Liang Chen, Fangyou Yu, Hong Zhang, Bingjie Wang

**Affiliations:** 1 Department of Clinical Laboratory, Shanghai Children’s Hospital, School of Medicine, Shanghai Jiao Tong University 667160, Shanghai, China; 2 Institute of Pediatric Infection, Immunity, and Critical Care Medicine, School of Medicine 12474, Shanghai, China; 3 Department of Clinical Laboratory, Shanghai Pulmonary Hospital, School of Medicine, Tongji University 12476, Shanghai, China; 4 School of Pharmacy and Pharmaceutical Sciences, University at Buffalo 15497, Buffalo, New York, USA; London School of Hygiene & Tropical Medicine, London, United Kingdom

**Keywords:** methicillin-resistant *Staphylococcus aureus*, genome sequencing, molecular epidemiology, children, antibiotics

## Abstract

The objective of this research was to investigate the genomic epidemiology of methicillin-resistant *Staphylococcus aureus* (MRSA) in a pediatric population in Shanghai, China. Whole-genome sequencing was conducted for 492 randomly selected MRSA isolates obtained from a pediatric hospital between 2013 and 2022. ST59 (37.4%), ST398 (22.4%), ST88 (5.7%), and ST22 (5.5%) were the predominant lineages among these children. While ST59 maintained a dominant annual proportion before 2017, the proportion of ST398 gradually increased from 2013 to 2016, with ST398 ultimately emerging as a prevalent clone with a proportion comparable to that of ST59 after 2017. Among the prevalent STs, the *spa*-SCC*mec* structure also experienced dynamic changes. Within ST59, the t437-IV subtype experienced a decline and has even been replaced by t172-IV in recent years. In ST398, the t011-V subtype appeared in 2014 and rapidly became the leading subtype. The antibiotic resistance profiles and virulence factors exhibited clone-related features. Compared with other prevalent lineages, ST59 presented high resistance to erythromycin and clindamycin, whereas ST398 presented relatively low resistance to common antimicrobial agents and fewer virulence determinants. Panton-Valentine leucocidin was more common in ST338 and ST1232, whereas toxic shock syndrome toxin was closely associated with ST1 and ST5. The MRSA cases could also be classified into community- and hospital-associated cases, with highly significant differences between the two in terms of demographic characteristics, clindamycin susceptibility, and virulence genes. In conclusion, this study revealed high genetic diversity and dynamic changes in the molecular epidemiology of pediatric MRSA isolates from Shanghai collected over a decade.

**IMPORTANCE:**

Methicillin-resistant *Staphylococcus aureus* (MRSA) has emerged as a significant global health concern. Previous research on MRSA epidemiology has predominantly focused on adult populations or targeted specific infection sites, while there was limited research on the long-term evolution of MRSA from the pediatric population. This study addresses this knowledge gap by conducting a comprehensive, 10-year surveillance of pediatric MRSA isolates using whole-genome sequencing. We characterized the molecular typing, as well as the phenotypic and genotypic antimicrobial resistance profiles, and virulence factors present in MRSA isolates obtained from children. Our results highlight the imperative for continuous, vigilant monitoring of MRSA within the pediatric demographic to track its evolving genetic landscape.

## INTRODUCTION

*Staphylococcus aureus* is recognized as one of the primary opportunistic bacterial pathogens affecting humans and is frequently implicated in conditions such as endocarditis, bacteremia, osteomyelitis, and skin and soft tissue infections (1). Multidrug resistance has emerged as a major health concern worldwide (2), with methicillin-resistant *S. aureus* (MRSA) posing a growing challenge since its initial identification in 1961 (3). In addition to being resistant to oxacillin (OXA) and β-lactam antibiotics, recent studies have indicated that MRSA can also exhibit resistance to vancomycin (VAN), linezolid (LNZ), and daptomycin (DAP), which are considered last-line treatment options (4). Moreover, MRSA is associated with considerable morbidity and mortality, largely attributable to its abundant repertoire of virulence determinants, such as biofilm formation, immune evasion, hemolysis, and enterotoxins (5).

MRSA was once confined largely to the healthcare environment. However, since the 1990s, there has been an explosion in the incidence of MRSA infections in healthy community dwellers without prior healthcare contact, which has been associated with the recognition of novel MRSA clones known as community-associated MRSA (CA-MRSA) (6). CA-MRSA strains were often susceptible to most non-β-lactam antimicrobial agents and displayed enhanced virulence (7), associated with distinct lineages of MRSA, such as ST59, ST8 (USA300), and ST22 (8, 9). Over time, these clones began invading healthcare settings, and the distinctions between CA-MRSA and hospital-associated MRSA (HA-MRSA) have become increasingly blurred. For example, there were increasing reports that USA300 had been a common nosocomial pathogen in America (10). A comprehensive understanding of HA-MRSA and CA-MRSA isolates is essential for the development of effective treatment and prevention of MRSA infections across diverse healthcare and community settings.

MRSA epidemics are characterized by the predominance of varied clonal types in different geographic regions (2). In Asia, sequence type (ST) 239, ST5, and ST22 are the most frequently reported, while ST8 and ST45 are prevalent in North America (11). However, the number of prevalent clones in a given area may change over time. Several studies have suggested that the CA-MRSA clone ST59 is gradually replacing the HA-MRSA clone ST239 in China (12, 13). More importantly, the potential for interspecies transmission underscores the significant capacity of MRSA to adapt to different hosts and environments (14). In particular, livestock-associated MRSA (LA-MRSA) has attracted increasing attention because it has been frequently reported in humans. Since 2005, ST398 has been well described as a predominant lineage of LA-MRSA in Europe (15), with subsequent reports of its presence in the human populations of China and the United States (16, 17).

To date, epidemiological research in China has focused mainly on the adult population and has targeted specific infection sites or patient wards (9, 18). However, there remains a lack of comprehensive understanding of the long-term evolution of MRSA derived from pediatric populations. In this study, we randomly collected a total of 492 MRSA isolates from a tertiary pediatric hospital in Shanghai between 2013 and 2022. We aimed to characterize the genetic background and identify predominant lineages of MRSA in pediatric patients.

## MATERIALS AND METHODS

### Clinical isolates and information collection

First, we collected data on methicillin-susceptible *S. aureus* and MRSA strains isolated from 2013 to 2022 in a pediatric hospital in Shanghai, China, to calculate the percentage of MRSA strains among *S. aureus* for each year. An MRSA isolate was defined as those carrying the *mecA* gene or exhibiting resistance to cefoxitin (FOX; MIC ≥8 µg/mL). Using the RANDBETWEEN randomization module in Microsoft Excel, we initially selected 50 isolates annually from 2013 to 2022. Eight isolates were excluded due to incomplete clinical information or the low quality of whole-genome sequencing outputs. Finally, a total of 492 non-duplicate MRSA clinical isolates were included in this study. The relevant clinical information, including sex, age, source, and clinical diagnosis, was extracted from the anonymized laboratory information system. The included patients were divided into six age groups: newborn (0–28 days), infant (1–12 months), toddler (13 months–3 years), preschool (4–7 years), school-aged (8–12 years), and adolescent (13–16 years). CA-MRSA and HA-MRSA were both defined based on the clinical criteria. CA-MRSA strains were isolated from outpatients or inpatients within 48 hours of hospital admission, without a history of hospitalization, long-term care facility, surgery, permanent indwelling catheter use, percutaneous medical device use, or positivity for MRSA culture in the previous 12 months. HA-MRSA strains were defined as those that did not meet the above CA-MRSA standards (19).

### Antimicrobial susceptibility testing

We conducted antimicrobial susceptibility testing via the broth microdilution method. The antibiotics tested included FOX, OXA, erythromycin (ERY), clindamycin (CLI), VAN, sulfamethoxazole/trimethoprim (SXT), LNZ, rifampicin (RIF), gentamicin (GEN), ciprofloxacin (CIP), tetracycline (TCY), fusidic acid (FA), mupirocin (MOP), teicoplanin (TCL), DAP, and dalbavancin (DAL). The interpretative criteria followed the CLSI guidelines (20), except for FA, for which EUCAST breakpoints were applied (21).

### Whole-genome sequencing and bioinformatic analyses

The total DNA of the MRSA isolates was extracted via a TlANamp Bacteria DNA Kit (TIANGEN) with additional lysostaphin. The isolated genomic DNA was sequenced on the Illumina NovaSeq platform in 2 × 150 bp paired-end mode. The raw data were filtered with fastp v0.20.1 (22) and then *de novo* assembled into contigs using CLC Genomics Workbench software (version 12.0; CLCbio). The N50 contig number was 55.28 ± 48.15, with 137.69 ± 44.89 kbp. Multilocus sequence typing (MLST) was performed via the PubMLST database (https://pubmlst.org/organisms/staphylococcusaureus). The *spa* and SCC*mec* types were predicted using SpaFinder and SCCmecFinder, respectively (23, 24).

Moreover, the virulence factor genes and antimicrobial resistance genes were identified by ABRicate v1.01 (https://github.com/tseemann/abricate) using the VFDB and CARD databases (25, 26). The candidate virulence genes were associated with adhesion, enterotoxins, immune evasion, secretion, toxins, hemolysis, serine protease, and iron uptake. The genes were annotated via Prokka v1.14.5 (27). For phylogenetic analyses, a core-genome alignment based on the concatenation of 1,702 core genes was obtained via Panaroo v1.5.0 (28) with default parameters. A maximum-likelihood phylogenetic tree was constructed via FastTree v2.1.11 (model GTR + GAMMA) with 1,000 bootstrap replicates (29). The tree was visualized using iTOL v6 (30).

### Statistical analyses

Statistical analyses were performed using GraphPad Prism 9.0. The data were analyzed using Pearson’s *χ*^2^ test, Fisher’s exact test, and Student’s *t* test, as appropriate. A *P* value of  <0.05 was considered to indicate statistical significance.

## RESULTS

### Molecular typing characteristics

In the present study, a total of 492 non-duplicated MRSA clinical isolates were collected over 10 years. Thirty-one distinct STs were identified, with ST59 (37.4%, 184/492), ST398 (22.4%, 110/492), ST88 (5.7%, 28/492), and ST22 (5.5%, 27/492) being the most prevalent (Table S1). Additionally, three isolates belonging to novel STs were identified, and their genome sequences have been submitted to PubMLST (https://pubmlst.org/organisms/staphylococcus-aureus/, id: 47326–47328), where they have been assigned to new STs (ST9203, ST9204, and ST9205). The identified STs can be classified into 11 clonal complexes (CCs), with CC59 (42.9%, 211/492), CC398 (23.6%, 116/492), and CC5 (7.7%, 38/492) being the most common CCs, collectively accounting for more than 70% of the isolates.

All the MRSA isolates were assigned to 77 types by *spa* typing, with 15 new types identified. ST88 dominated among the novel *spa* types, accounting for 40% (6/15) of cases. The *spa* types of the MRSA isolates exhibited a strong correlation with their CCs. The predominant *spa* type was t437 (24.2%, 119/492), which was the major type within CC59 (56.4%, 119/211). The second most common type was t011 (12.6%, 62/492), classified under CC398. Within CC59, t172 was also frequently observed (20.4%, 43/211). Notably, the ST59 lineage presented a greater diversity of *spa* types than the other CC59 clones did (Table S2).

Four SCC*mec* types were identified, namely, II, III, IV, and V. SCC*mec* IV (57.5%, 283/492) was the most prevalent, followed by the SCC*mec* V cassette (36.4%, 179/492). Twenty-four isolates could not be classified into any SCC*mec* type, with nearly one-third of them belonging to ST88 (28.6%, 8/28). CC59 and CC398 were closely correlated with SCC*mec* IV (85.3%, 180/211) and SCC*mec* V (96.6%, 112/116), respectively. Notably, ST338, a variant clone of ST59, was classified under SCC*mec* V. Additionally, SCC*mec* IV was subdivided into IVa, IVc, IVg, and IVi. CC59 exhibited a strong association with SCC*mec* IVa (84.4%, 178/211), whereas CC88 was more likely to be associated with SCC*mec* IVc (67.9%, 19/28; Table S2).

In general, the population structure of the MRSA isolates in our study was dominated by ST59-t437-IV (17.3%, 85/492), ST398-t011-V (11.4%, 56/492), and ST59-t172-IV (7.1%, 35/492).

### Demographic characteristics of the MRSA isolates

**Fig 1:**
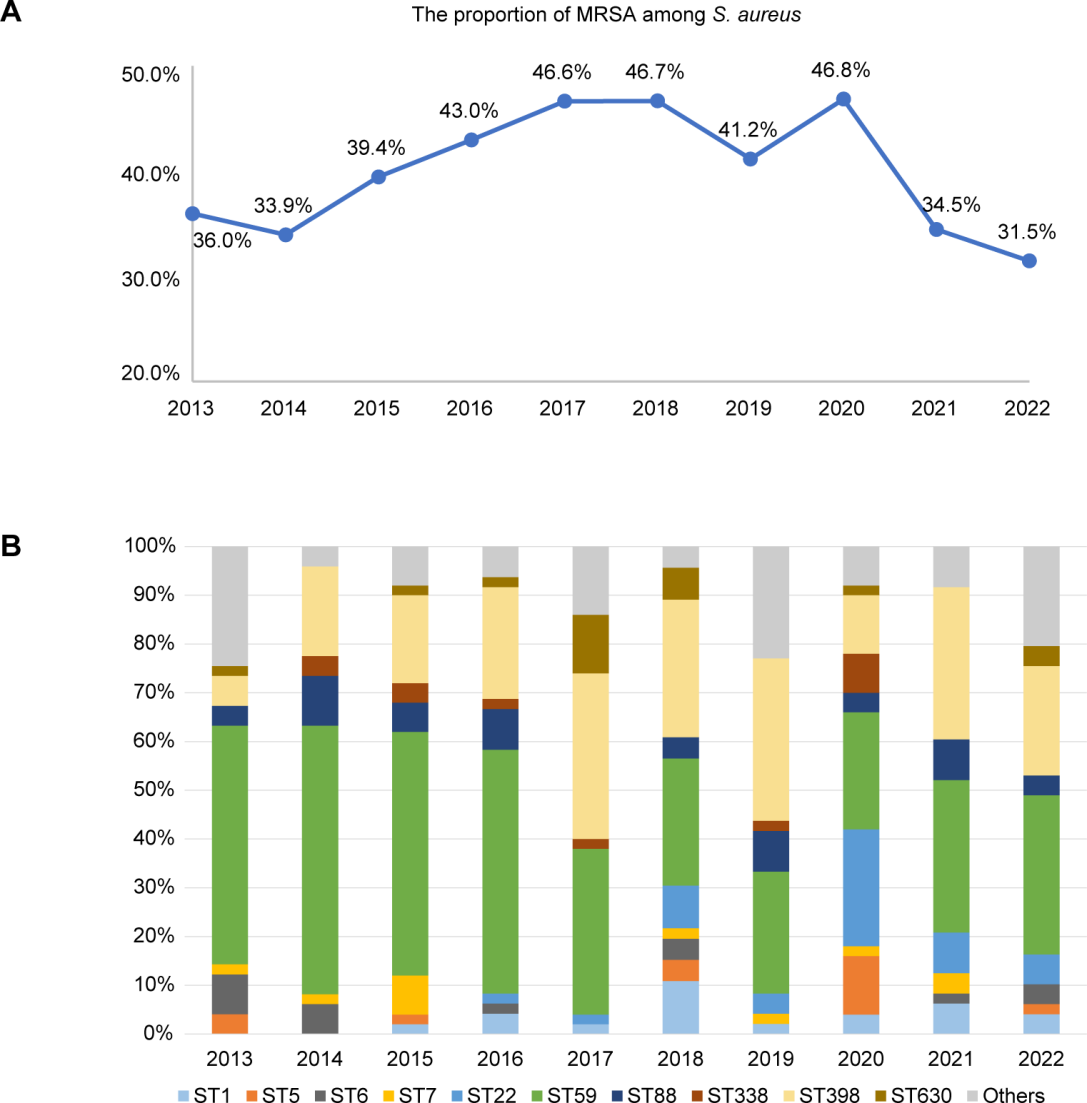
(**A**) The proportion of MRSA among *S. aureus* in each year. (**B**) The annual proportion of each ST. STs with <10 isolates were included in the “others” group.

Among the 492 MRSA isolates, a majority were collected from children under 1 year of age (61.0%, 300/492). The sample sources of isolates varied widely and included sputum, pharyngeal swabs, pus, secretions, blood, cerebrospinal fluid, and central venous catheters. Sputum was the predominant sample source (60.6%, 298/492), especially among neonates and children under 3 years of age. The most frequently identified clones from the sputum samples were ST59 and ST398. For the second most common source, pus (22.0%, 108/492), isolates were more frequently obtained from school-aged individuals and adolescents, with ST59 and ST22 being the most common clones in this group. Moreover, in our study, several isolates were derived from vaginal secretions, predominantly within the preschool subgroup (65.0%, 13/20; Table S2). The percentage of MRSA strains among *S. aureus* was slightly higher in male patients, constituting 58.5% (288/492) of the total. The annual proportions of MRSA among *S. aureus* tended to increase from 2014 to 2018, corresponding with an increase in the prevalence of ST398. Nevertheless, a significant decline in this rate was observed in 2021 (34.5% vs 46.8%, *χ*^2^ test, *P* < 0.001; Fig. 1A).

On the basis of the clinical data, the MRSA isolates were categorized into CA-MRSA (23.4%, 115/492) and HA-MRSA (76.6%, 377/492). Statistical analysis revealed no significant differences in the annual proportions of CA-MRSA and HA-MRSA (Table 1, χ test, *P* = 0.33). HA-MRSA was more commonly detected in children under 1 year of age, whereas CA-MRSA was more prevalent among preadolescent children in this study. HA-MRSA was predominantly isolated from the neonatology ward, whereas CA-MRSA was significantly more common in the pulmonology ward (Table 2).

**TABLE 1:** The yearly demographic characteristics of the samples involved in this research between 2013 and 2022 (%)

	2013 (*n* = 50)	2014 (*n* = 49)	2015 (*n* = 50)	2016 (*n* = 49)	2017 (*n* = 50)	2018 (*n* = 47)	2019 (*n* = 48)	2020 (*n* = 50)	2021 (*n* = 49)	2022 (*n* = 50)
Specimen source										
Sputum	46.0	69.4	90.0	73.5	58.0	55.3	66.7	40.0	57.1	50.0
Pus	22.0	16.3	8.0	16.3	24.0	19.2	14.6	48.0	26.5	24.0
Swab	4.0	4.1	0.0	4.1	4.0	10.6	14.6	4.0	6.1	6.0
Secretion	20.0	4.1	0.0	2.0	4.0	2.1	2.1	0.0	4.1	2.0
Blood/cerebrospinal fluid	2.0	4.1	0.0	0.0	0.0	4.3	0.0	2.0	4.1	12.0
Others	6.0	2.0	2.0	4.1	10.0	8.5	2.1	6.0	2.0	6.0
Epidemiological classification										
CA-MRSA	22.0	16.3	14.0	24.5	20.0	19.1	41.7	26.0	24.5	26.0
HA-MRSA	78.0	83.7	86.0	75.5	80.0	80.9	58.3	74.0	75.5	74.0

**TABLE 2:** Demographic characteristics of CA-MRSA and HA-MRSA cases, *n* (%)

	CA-MRSA (*n* = 115)	HA-MRSA (*n* = 377)	*P*-value
Sex			
Male	66 (57.4)	222 (58.9)	>0.05
Female	49 (42.6)	155 (41.1)	>0.05
Age			
0–28 days	1 (0.9)	105 (27.9)	<0.001
1–12 months	36 (31.3)	159 (42.2)	<0.05
13 months to 3 years	31 (27.0)	59 (15.9)	<0.05
4–7 years	27 (23.5)	22 (5.8)	<0.001
8–12 years	18 (15.7)	25 (6.6)	<0.05
13–16 years	2 (1.7)	6 (1.6)	>0.05
Specimen source			
Sputum	37 (32.2)	261 (69.2)	<0.001
Pus	44 (38.3)	64 (17.0)	<0.001
Swab	11 (9.6)	17 (4.5)	<0.05
Secretion	12 (10.4)	8 (2.1)	<0.05
Blood/CSF^*a*^	5 (4.3)	9 (2.4)	>0.05
Others	6 (5.2)	18 (4.8)	>0.05
Outpatient department			
Otorhinolaryngology	10 (8.7)	3 (0.8)	<0.05
Dermatology	7 (6.1)	0	<0.05
Others	11 (9.6)	8 (2.1)	<0.05
Inpatient department			
Neonatology	5 (4.3)	156 (41.4)	<0.001
PICU^*a*^	5 (4.3)	29 (7.7)	>0.05
Pulmonology	25 (21.7)	27 (7.2)	<0.05
General surgery	19 (16.5)	14 (3.7)	<0.05
Orthopedic	13 (11.3)	1 (0.3)	<0.05
Others	20 (17.4)	139 (36.9)	<0.05

^*a*^ CSF, cerebrospinal fluid; PICU, pediatric intensive care unit.

### Dynamic evolution of MRSA from 2013 to 2022

**Fig 2:**
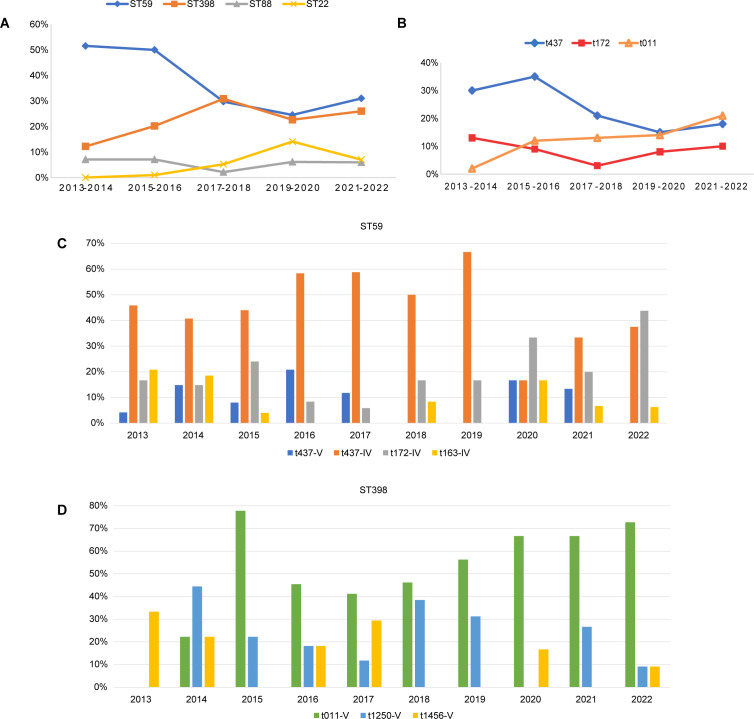
The changing molecular characteristics by year. (**A**) The 2-year temporal changes of four predominant STs. (**B**) The 2-year temporal changes of three main *spa* types. (**C**) The proportion of major molecular structures of ST59 during the 10 years. (**D**) The proportion of major molecular structures of ST398 during the 10 years.

The prevalence of different molecular types over the 10 years showed dynamic fluctuations (Fig. 1B). From 2013 to 2016, ST59 maintained a dominant position in terms of its annual proportion. However, its proportion started to decline in 2017 (from a maximum of 55.1% to a minimum of 24.0%). In contrast, ST398 exhibited a consistent increase from 2013 to 2017, with the proportion rising from 6.0% to 34.0%. Subsequently, the prevalence of ST398 either matched or exceeded that of ST59, except in the year 2020. ST88 exhibited a relatively stable distribution throughout the study period. Notably, ST22 emerged in 2016, and its proportion sharply increased in 2020, reverting to the previous levels in 2021 (Fig. 2A). The *spa* types were conserved within their respective CCs, yet they also underwent notable changes over the decade (Fig. 2B). The proportion of the predominant type t437 slightly increased in the initial years, followed by a gradual decline until 2022. Conversely, the prevalence of t011 showed a persistent increase, starting from a low value and ultimately reaching parity with t437, which aligns with the trends observed for their associated lineages ST59 and ST398, respectively. The prevalence of another *spa* type of CC59, t172, initially displayed a declining trend in the early years but has increased again in recent years (Table S3; Fig. 2B). Further analysis of the two predominant clones, ST59 and ST398, revealed temporal dynamics in their population structures over the 10-year period. For ST59, the t437-IV subtype was the most dominant subtype from 2013 to 2019, but in recent years, its proportion within ST59 has decreased, with t172-IV emerging as the dominant subtype in 2020 and 2022 (Fig. 2C). For ST398, the leading type, t011-V, was first identified in 2014, and it rapidly became dominant beginning in 2015. The isolation frequency of t1250-V fluctuated from 2014 to 2022 (Fig. 2D).

**Fig 3:**
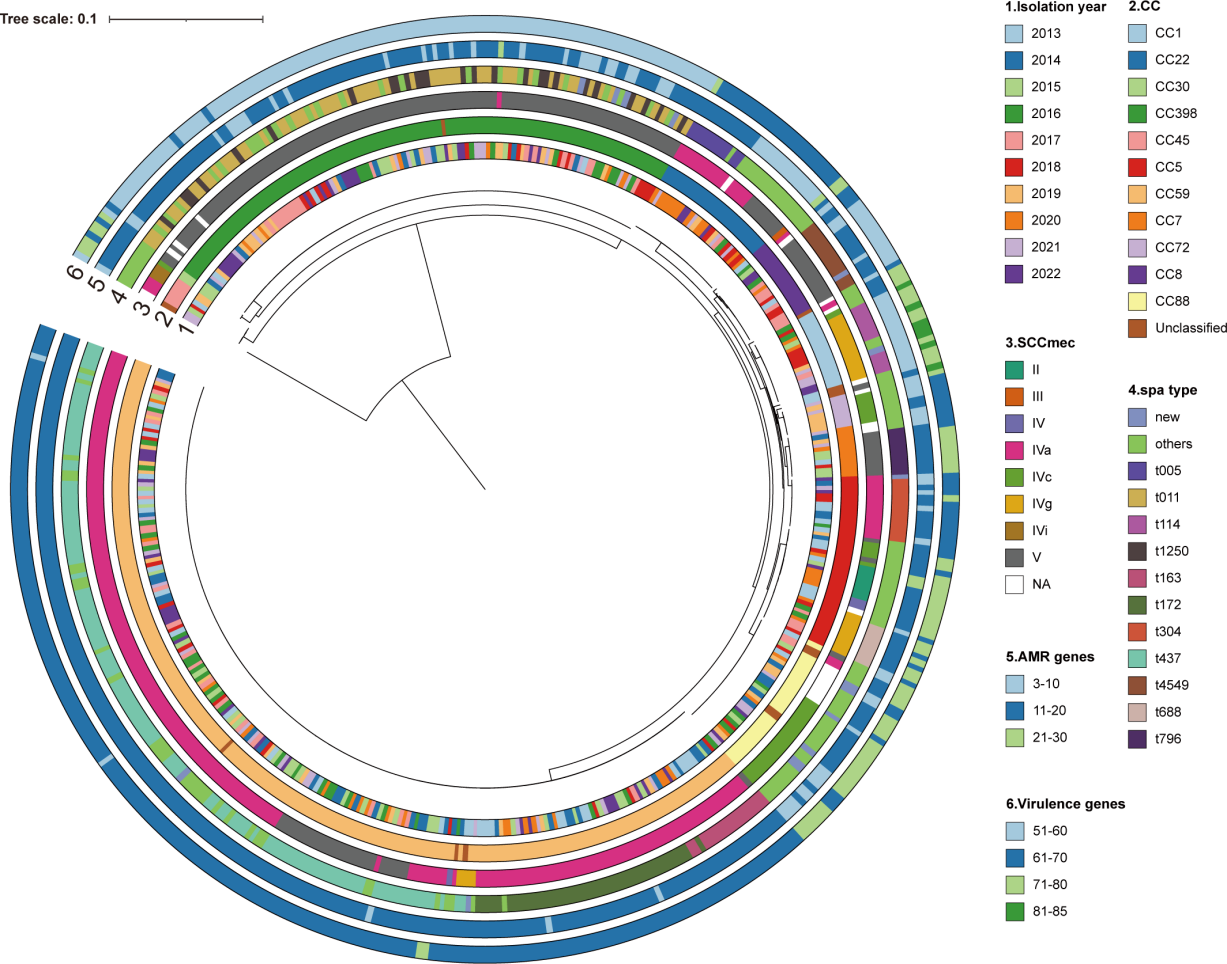
Phylogenetic tree of all the 492 MRSA. Isolation year, CC types, SCC*mec* types, *spa* types, the carrying number of antimicrobial resistance genes (AMR), and virulence genes are color coded in the outside rings.

We constructed phylogenetic trees for all 492 MRSA isolates to determine their evolutionary relationships. The analysis revealed the genetic diversity of each clone over the 10-year period and their evolutionary relationships. The CC22 strains (ST22) isolated in 2020 clustered together, which was consistent with their rapid dissemination in 2020. Notably, several ST398 strains isolated in 2017 were also closely related, indicating their evolution from a common ancestor (Fig. 3).

It is important to highlight the atypical epidemic trends observed in 2020. First, the annual percentage of MRSA strains among *S. aureus* peaked in 2020 and then plunged immediately in 2021 (Fig. 1). Furthermore, the decline in ST398 abundance interrupted a previously established upward trend, although this ST experienced a resurgence in 2021. In contrast, the abundance of ST22 increased clearly in 2020 and then reverted (Fig. 1B). Interestingly, sputum samples consistently represented a majority of the samples collected each year, but the number of isolates derived from pus exceeded that derived from sputum in 2020. We subsequently collected annual data on the total number of pus and upper respiratory tract samples (except for 2013). The analysis revealed an increase in the ratio of pus/upper respiratory tract samples in 2020, mainly due to a decrease in upper respiratory tract samples, while the number of pus samples stayed stable (9.1% vs 5.7% ± 0.71%, Student’s *t* test, *P* < 0.05). The reduction in upper respiratory tract samples may be attributed to non-pharmaceutical interventions during the COVID-19 outbreak (31, 32). This may account for the shift in the percentage of MRSA isolates originating from pus (Table S4).

### Phenotypic and genotypic antimicrobial susceptibility

**Fig 4:**
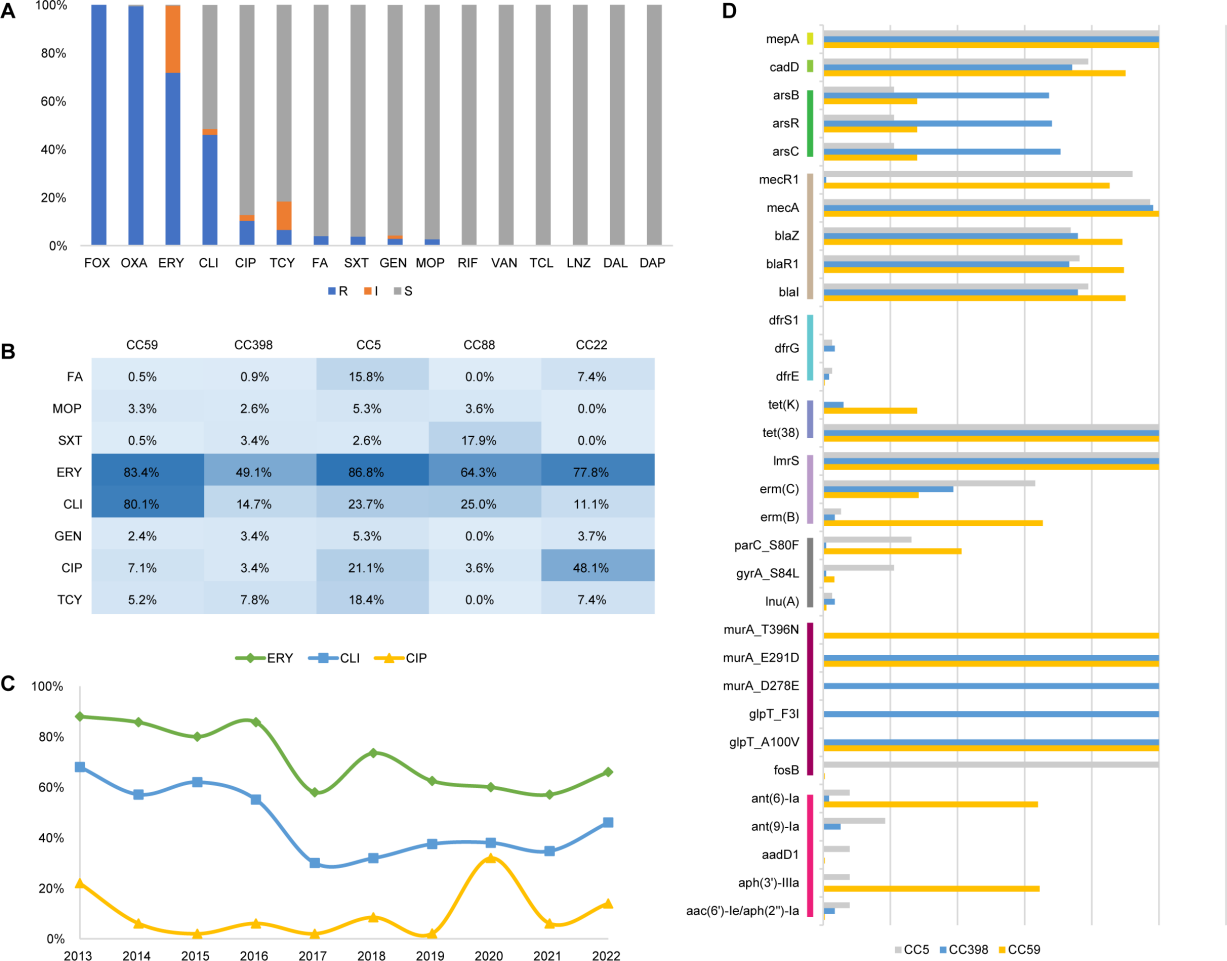
Phenotypic and genetic antimicrobial susceptible characteristics. (**A**) The overall rates of antibiotic resistance (R), intermediate susceptibility (I), and susceptibility (S). (**B**) The resistance rate of eight antibiotics in dominant lineages. (**C**) Annual trends in resistance rates for ERY, CLI, and CIP. (**D**) The antimicrobial resistance genes carried by CC59, CC398, and CC5.

The phenotypic antimicrobial susceptibility profile of the MRSA isolates was determined using the broth microdilution method (Fig. 4; Fig. S1). All of the MRSA isolates were resistant to cefoxitin, and as expected, a majority of the strains harbored multiple β-lactam resistance genes, including *blaI*, *blaR1*, *blaZ*, *mecA*, and *mecR1* (Table S5). A total of 97.8% (480/492) of the MRSA strains carried *mecA*. Notably, three *mecA*-positive isolates exhibited susceptibility to oxacillin. A high rate of erythromycin resistance (71.8%) was observed, particularly within CC5 (86.8%) and CC59 (83.4%), which corresponds with the relatively high carriage rates of *erm(C)* in CC5 and *erm(B)* in CC59. Clindamycin resistance was more common in CC59 (80.1% vs 11.1%–25.0%, *χ*^2^ test, *P* < 0.001). However, the direct lincosamide resistance genes *lnu(A)*, *lnu(G)*, and *lsa(A)* were infrequently identified across all the isolates, while the macrolide-associated gene *ermB* affecting susceptibility to clindamycin had a relatively high prevalence within the CC59 lineage (65.4%, 138/211; Table S5). In contrast, CC398 presented a relatively low level of resistance to erythromycin and clindamycin (Fig. 4B). Therefore, the rates of resistance to both antibiotics have declined since 2017 (Fig. 4C). Although the overall resistance to ciprofloxacin in this study was low (10.3%), it was significantly greater in CC22 than in other prevalent lineages (48.1% vs 3.6%–21.1%, *χ*^2^ test, *P* < 0.001), which was attributed to more frequent mutations in *gyrA_S84L* and *parC_S80F* in CC22. This trend was further reflected by an increased rate of resistance to ciprofloxacin in 2020 (Fig. 4C). The MRSA isolates in this study overall demonstrated low resistance rates to fusidic acid (3.9%), mupirocin (2.6%), sulfamethoxazole/trimethoprim (3.7%), and gentamicin (2.8%). However, ST630 may tend to be resistant to fusidic acid, as 86.7% of the strains carried *fusB*. Importantly, none of the isolates were resistant to vancomycin, linezolid, rifampicin, teicoplanin, daptomycin, or dalbavancin. The tetracycline-related gene *tet (38)* and the efflux-related gene *mepA* were identified in all the examined isolates (Fig. 4D; Table S5). Notably, a statistically significant difference in resistance rate to clindamycin was observed between CA-MRSA and HA-MRSA (Table 3).

**TABLE 3:** Comparative antibiotic resistance of CA-MRSA and HA-MRSA isolates, *n* (%)

	CA-MRSA (*n* = 115)	HA-MRSA (*n* = 377)	*P*-value
FA	6 (5.2)	13 (3.4)	>0.05
MOP	4 (3.5)	9 (2.4)	>0.05
SXT	4 (3.5)	14 (3.7)	>0.05
ERY	88 (76.5)	265 (70.3)	>0.05
CLI	62 (53.9)	165 (43.8)	<0.05
GEN	3 (2.6)	11 (2.9)	>0.05
CIP	9 (7.8)	41 (10.9)	>0.05
TCY	9 (7.8)	23 (6.1)	>0.05

### Virulence factors vary among clones

Diverse virulence genes enable MRSA to invade and adapt to humans. A total of 134 virulence factors were examined (Table S6). Among them, 49 virulence genes associated with various virulence characteristics presented carriage rates exceeding 90%, indicating a high level of conservation in MRSA. Compared with other prevalent clones, CC398 carried fewer virulence genes, whereas CC1 harbored more virulence factors (Fig. 3; Table S6). In our cohort, 16.1% of the MRSA isolates carried *lukF-PV* and *lukS-PV*, which encode Panton-Valentine leucocidin (PVL), a well-studied toxin in *S. aureus* and a molecular marker in CA-MRSA (33). Indeed, its carriage rate was significantly higher in CA-MRSA in the present study, at 33.9% (Table 4). Notably, all the ST338 and ST1232 isolates were positive for PVL, and all the CC5 strains were PVL negative. The carriage rate of PVL in ST59-t437-V was significantly higher than that in other subtypes of ST59 (85.7%, 12/14; *χ*^2^ test, *P* < 0.001).

**TABLE 4:** Virulence genes carried by CA-MRSA and HA-MRSA isolates, *n* (%)

	CA-MRSA (*n* = 115)	HA-MRSA (*n* = 377)	*P*-value
*pvl*	39 (33.9)	40 (10.6)	<0.001
*tsst-1*	8 (7.0)	32 (8.5)	>0.05
*chp*	105 (91.3)	320 (84.9)	<0.05
*sak*	92 (80.0)	344 (91.2)	>0.05
*scn*	109 (94.8)	348 (92.3)	>0.05
*sea*	20 (17.4)	100 (26.5)	<0.05
*sep*	6 (5.2)	40 (10.6)	<0.05
*coa*	107 (93.0)	304 (80.6)	<0.05
*vWbp*	10 (8.7)	46 (12.2)	>0.05
*clfA*	48 (41.7)	142 (37.7)	>0.05
*clfB*	30 (26.1)	135 (35.8)	<0.05
*cna*	2 (1.7)	10 (2.7)	>0.05
*fnbA*	75 (65.2)	277 (73.5)	>0.05
*fnbB*	68 (59.1)	231 (61.3)	>0.05
*sdrD*	55 (47.8)	201 (53.3)	>0.05
*sdrE*	82 (71.3)	262 (69.5)	>0.05

**Fig 5:**
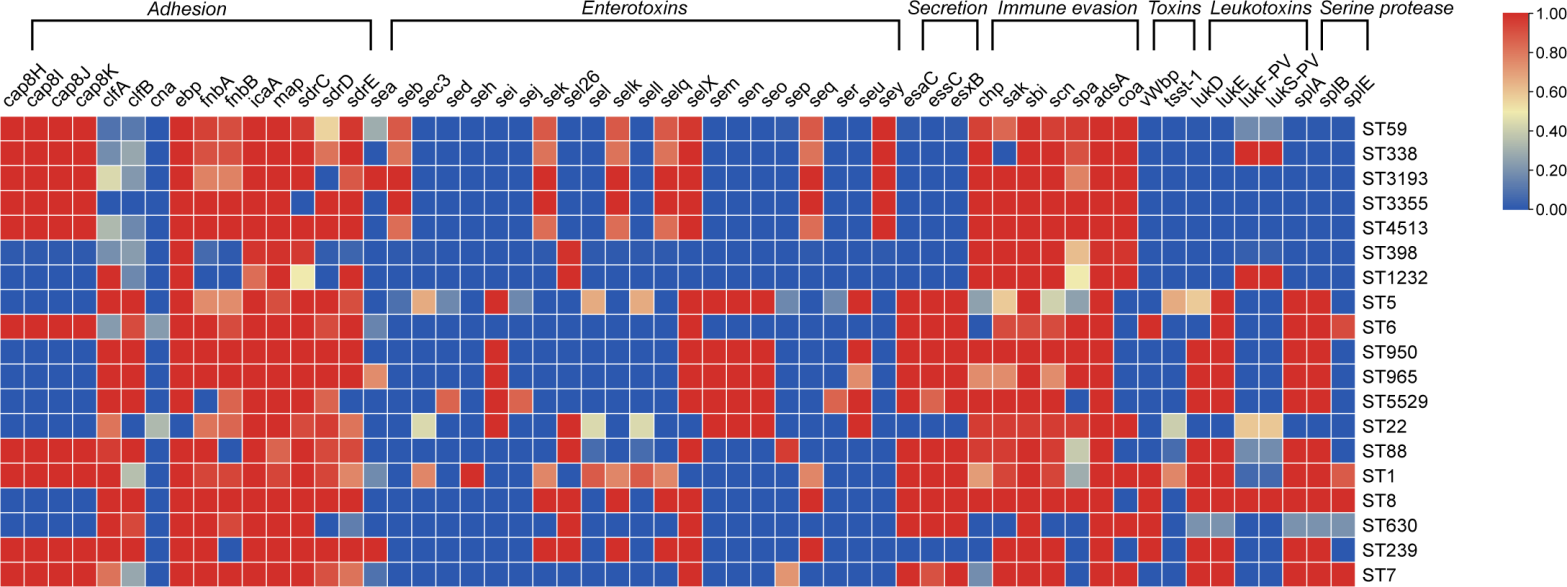
The distribution of virulence determinants. Virulence genes with >99% or 0% carriage rate were not displayed. More details can be found in Table S6.

Overall, most of the detected virulence genes presented clone-related features. The toxic shock syndrome toxin (TSST) encoded by *tsst-1* was frequently detected in ST1 (76.5%, 13/17) and ST5 (66.7%, 8/12). Moreover, more than 80% of ST22 isolates harbored either *pvl* or *tsst-1*, with all TSST-positive ST22 isolates belonging to SCC*mec* IV. The adhesion-related virulence gene *cna* was absent in most clones, except ST22. In contrast, the *ebp* gene was absent in ST22, whereas it was detected in nearly all the other clones. The frequency of biofilm-associated genes was generally high in all the MRSA strains, except for ST398 isolates, which largely lacked *fnbA*, *fnbB*, *sdrD*, and *sdrE* (Fig. 5). The secretion system-associated genes *esaC*, *essC*, and *esxB* were all absent in CC59, CC398, and CC22. Additionally, genes of the immune evasion cluster (IEC), including *scn*, *chp*, *sea*, *sep*, and *sak*, exhibited an ST-related distribution. The gene *sak* was prevalent in CC59, except in ST338. A majority of the MRSA isolates exhibited an *scn-chp-sak* pattern, except ST630, which did not carry the IEC. Enterotoxins also exhibited variability among strains. A majority of CC59 isolates harbored *seb-sek-selk-selq-seq-sey*; however, most isolates of subclone CC59-t437 lacked these enterotoxins. Similarly, the *sea* was absent in CC59-t437 but was universally present in CC59-t163 and CC59-t172, suggesting a difference in virulence associated with *spa* type (Fig. 5).

## DISCUSSION

In the present study, 492 MRSA isolates were randomly collected from a single pediatric cohort over the past decade to investigate the epidemiology and evolution of MRSA in children. The included samples covered a wide range of sources and were collected from individuals aged 0–16 years.

The phylogenetic analysis revealed high genetic diversity of the MRSA isolates across 10 years. During the research period, CC59 emerged as the predominant lineage, with the Asian-Pacific clone ST59-t437-IV being the most prevalent sub-lineage. This finding aligns with previous studies conducted on children in Beijing and Sichuan, as well as on adults in other regions of China (34–37). Notably, the temporal patterns of prevalence varied between children and adults. In an all-age and multicenter study on Chinese MRSA, the prevalence of ST59 increased from 27.8% to 35.6% between 2014 and 2020 (9). In contrast, we found a decline in ST59 prevalence from 55% to 24% over the same 6-year period.

Accordingly, the prevalence of ST398 steadily increased from 2013 to 2017, after which both ST398 and ST59 became the predominant lineages. This dissemination capacity and the prevalence trends suggest that ST398 may have strong host or environmental adaptations (1). In recent years, an obvious increase in the prevalence of ST398 has been observed in adults (35, 38). However, as early as 2013, the prevalence of ST398 in children increased gradually. Notably, unlike ST398-t034, which is disseminated among adults (34), our study revealed that the prevalent subtype in the pediatric population was ST398-t011. Another study on epidemic clones in China revealed that ST59 and ST398 had greater virulence potential than their counterparts ST5 and ST239 (39). However, our findings revealed that ST398 presented relatively low carriage of adhesion virulence genes, minimal levels of enterotoxins, and almost no *pvl* or *tsst-1*, indicating a complex correlation between the virulence phenotype and gene expression and regulation. ST59 and ST398 are more strongly associated with SCC*mec* IV and V, which are smaller cassettes that can reduce the potential fitness burden (40). This may have contributed to their pandemic characteristics.

ST22 was the second most common type among the pus-derived isolates in the present study. Similarly, another study from Shanghai reported that MRSA isolates from bone and joint infections in children were also characterized, with ST22 being the second major genotype (41). MRSA ST22 reportedly exhibits high virulence and has strong potential to replace other previously epidemic MRSA clones, which can enable ST22 clones to cause more severe infections.

ST88 has been identified as a predominant circulating clone within both hospital and community settings in African countries (42) and has been sporadically reported in the food industry (43). One-third of the non-typeable SCC*mec* elements belonged to ST88. Evidence suggested that Chinese ST88 isolates were identified with lineage-specific pseudo-SCC*mec* genes and lacked *ccr* genes (44), which may explain the challenges encountered in SCC*mec* typing. Intriguingly, ST88 was the most prevalent among the newly identified *spa* types, with the principal types (t1764 and t3622) showing differences from previously reported types (t690) in adults (45).

ST5 MRSA strains have been described as representative emerging epidemic clones in East China, including Shanghai (35, 38). Nonetheless, the findings of our study revealed a different epidemiological pattern, as ST5 accounted for only 2.4%, indicating a divergence in the prevalence of ST5 between children and adults. ST239 was previously demonstrated to be the predominant HA-MRSA type in adults in China (12). Interestingly, despite the observed replacement of ST239 by ST5 and ST59 in adults since 2013 (46, 47), only one ST239 MRSA isolate was detected in our study in 2013, indicating an earlier or more pronounced decrease in the incidence of ST239 in children. In addition to the epidemic clones, some single STs were detected. ST4513, belonging to CC59, was previously reported in an endocarditis patient (48). However, ST5529 (CC5), ST6309, and ST4083 have not been previously reported.

The unusual epidemic trend in 2020, which varied from that in other years, can be linked to the emergence of the COVID-19 pandemic during that year. Another pediatric study in Henan, China, revealed that the *S. aureus* positivity in the respiratory system significantly decreased during the COVID-19 pandemic, whereas there were no obvious changes among isolates from non-respiratory system samples (49). This finding parallels the reduction in the number of upper respiratory tract samples noted in our study, which influenced the proportion of MRSA isolated from pus samples.

With respect to virulence determinants, the prevalence of PVL was 16.1%, similar to the reported rate of 15.1% among children in Southwest China (37). These data indicate that the rate of PVL in MRSA isolates from children is still low. Furthermore, PVL was more common in ST59-V than in ST59-IV, which is consistent with the previously reported ST59/SCC*mec* V*/*PVL-positive Taiwan clone (50).

In antimicrobial susceptibility testing, we identified three oxacillin-susceptible MRSA (OS-MRSA) isolates that carried the *mecA* gene. Previous studies have indicated that the prevalence of OS-MRSA in Shanghai is 1.8%, with most isolates derived from pediatric inpatients (51). Nucleotide substitutions within the *mecA* promoter (52), frameshift mutations in *mec*A (53), a loss of penicillin-binding protein 4 (54), and a deletion in the *blaR1* gene (55) are all possible mechanisms underlying the low-level oxacillin resistance of the OS-MRSA strains identified. There is evidence that CA-MRSA clone ST59 is more susceptible to antibiotics than the prevalent HA-MRSA clones ST239 or ST5 are (35). Notably, in our study, ST398 exhibited greater antibiotic susceptibility than ST59. Moreover, although it has been reported that CA-MRSA is more likely than HA-MRSA to be susceptible to CIP, CLI, GEN, and SXT (56), our data revealed no significant difference in resistance between CA-MRSA and HA-MRSA.

In conclusion, the molecular epidemiology of pediatric MRSA isolates in Shanghai experienced great dynamic changes during the 10-year period. Compared with the findings of previous studies on adults, there were many differences in the MRSA isolates between adults and children. Our study provides updated insights into pediatric MRSA, including clinical demographic features, molecular types, antibiotic profiles, resistance and virulence genes, and temporal evolution, highlighting the need for long-term surveillance of pediatric MRSA.

## Data Availability

The raw sequence data for 492 MRSA isolates have been deposited in the Sequence Read Archive under BioProject accession no. PRJNA1211827.

## SUPPLEMENTAL MATERIAL

Fig. S1 msystems.00371-25-s0001.docx The MIC distribution of 16 tested antibiotics in this study.

Supplemental Tables msystems.00371-25-s0002.xlsx Tables S1 to S6.

ASM does not own the copyrights to Supplemental Material that may be linked to, or accessed through, an article. The authors have granted ASM a non-exclusive, world-wide license to publish the Supplemental Material files. Please contact the corresponding author directly for reuse.
