# Supplementary material for: Longitudinal surveillance of the molecular evolution of methicillin-resistant Staphylococcus aureus isolates from pediatric patients in Shanghai, China, from 2013 to 2022
Source: mSystems. 2025 Apr 30;10(5):e00371-25. doi: 10.1128/msystems.00371-25 (PMC12090807; doi:10.1128/msystems.00371-25)
Supplement: Fig. S1 — The MIC distribution of 16 tested antibiotics in this study. [file msystems.00371-25-s0001.docx]

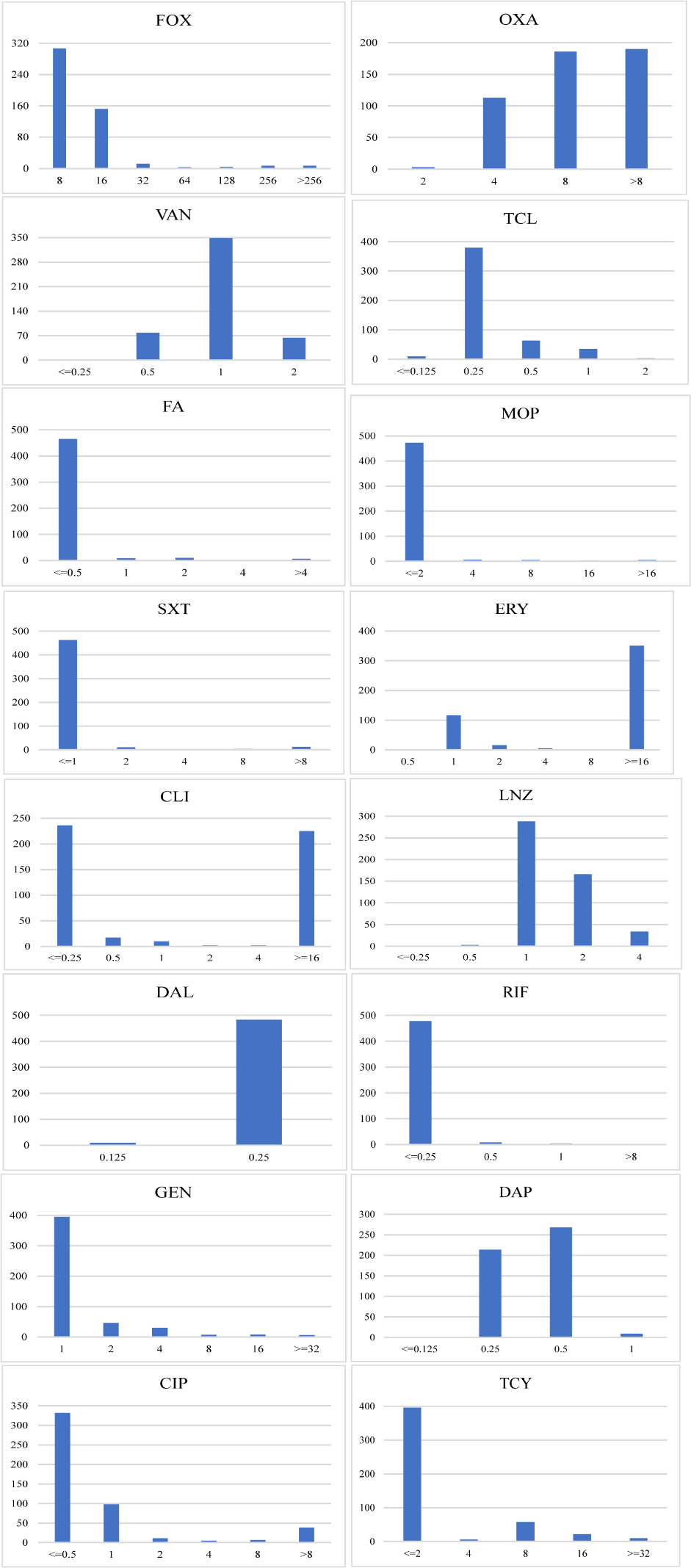


Figure S1. The MIC distribution of 16 tested antibiotics in this study. FOX: cefoxitin, OXA: oxacillin, VAN: vancomycin, TCL: teicoplanin, FA: fusidic acid, MOP: mupirocin, SXT: sulfamethoxazole/trimethoprim, ERY: erythromycin, CLI: clindamycin, LNZ: linezolid, DAL: dalbavancin, RIF: rifampicin, GEN: gentamicin, DAP: daptomycin, CIP: ciprofloxacin, TCY: tetracycline.
